# Description of a new species of the genus *Glenea* from Tibet, China (Coleoptera, Cerambycidae, Lamiinae, Saperdini)

**DOI:** 10.3897/zookeys.216.3360

**Published:** 2012-08-21

**Authors:** Meiying Lin, Li Dai

**Affiliations:** 1 Key Laboratory of Zoological Systematics and Evolution, Institute of Zoology, Chinese Academy of Sciences, Beichen West Road, Chaoyang Dist., Beijing, 100101, China; 2 Shanghai Entomological Museum, Chinese Academy of Science, Shanghai 200032, China

**Keywords:** New species, taxonomy, Oriental region

## Abstract

A new species, *Glenea jini* **sp. n.** is described from Tibet, China. It can be separated from other species of the genus *Glenea* Newman by the complicated black and ochre markings as well as characters of the genitalia.

## Introduction

In the progress of our research project on the “Study on the Systematics of Saperdini from China and the adjacent areas (Coleoptera: Cerambycidae: Lamiinae)”, many Chinese saperdine specimens have been recently collected as well as loaned from institutions, museums or private collections. In these collections two years ago, we found a noticeable new species from Tibet belonging to the genus *Glenea*. Since only male specimens were known, we were waiting for female specimens before officially publishing. Fortunately, during the first author’s visit to Shanghai Entomology Museum, some female specimens were found in their collection, and one fresh female was collected in August 2011 by a friend. In the current work, we describe this new species with detailed features of both the male and female genitalia.

Type depositories are abbreviated as follows:

CCCC Collection of Chang-chin Chen, Tianjin, China

IZAS Institute of Zoology, Chinese Academy of Sciences, Beijing, China

SHEM Shanghai Entomology Museum, Chinese Academy of Sciences, Shanghai, China

## Results

### Glenea jini sp. n.

urn:lsid:zoobank.org:act:81EB4C33-7179-4CF6-8993-A02AABCA3EF9

http://species-id.net/wiki/Glenea_jini

Figs 1 –8

#### Description.

**Figures 1–3.**
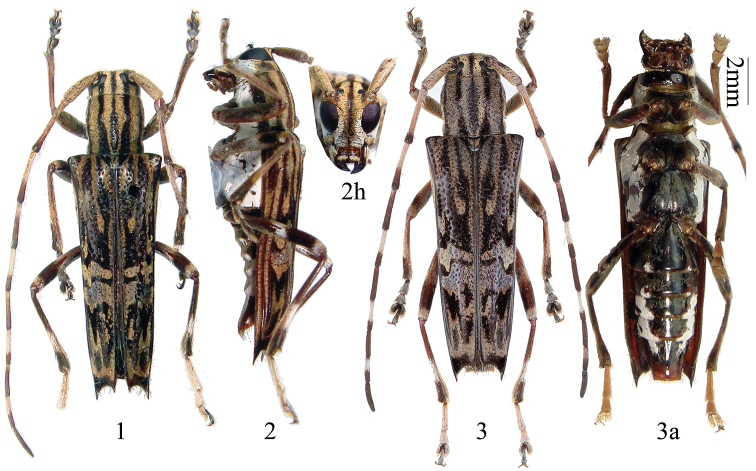
Habitus, *Glenea jini* sp. n. **1–2** male, from Tibet, China **1** holotype, IOZ(E)1859443 **2** paratype, IOZ(E)1859444. 2h. head, frontal view **3** paratype, female, from Tibet, China, photo by Wenxuan Bi. 3a. ventral view. Scale 2 mm.

Male (Figs 1–2): length: 11.5–14.0 mm, humeral width: 2.5–3.9 mm. Female (Figs 3, 3a): length:12.0–15.0 mm, humeral width: 3.0–4.5 mm. Body dark brown to black, all with thick pubescence except ventral medial part. The pubescence in dorsal view is black and ochre, forming quite complicated markings on head, pronotum and elytra. The pubescence in ventral view is white. Head (dark brown to black Fig. 2h); frons with ochre pubescence, with white pubescence along eyes (only lower half) and from genae to clypeus; pubescence of genae and temple white with ochre; vertex with two parallel ochre stripes and one black stripe between upper eye lobes; behind eyes striped with vittae of black, ochre, black, ochre and finally white (the white pubescence surrounding lower half of inferior eye lobes); those vittae matching with the vittae of prothorax except one additional black vitta before white ventral pubescence on sides of prothorax. Antennae reddish brown mixing with black, scattered with black bristles on undersides of 1^st^ to 8^th^ segments; tips of 3^rd^, 4^th^, 6^th^, 8^th^, 10^th^ and more than half apicalend of 5^th^, 7^th^, 9^th^ segments, and the whole of 11^th^ segment black, other parts covered with ochre and white pubescence. Prothorax with a medial black narrow stripe, then 6 stripes with alternating color of ochre and black on each side; prosternum with white pubescence, which extends to procoxal cavity. Scutellum black with ochre pubescence. Elytra black with complicated pubescent ochre stripes or vittae (Figs 1, 3): each elytron provided with three longitudinal stripes starting from the base, of which the middle one is the shortest, only reaching 3/10^ths^ of elytron length; two transverse wavy vittae just behind midpoint; apical 2/5^ths^ somewhat half black half ochre, with black apex. Sides of elytron covered with ochre pubescence except for the ridges and a black vitta after humerus (Fig. 2). Ventral surface mostly dark brown to black, with sides covered with dense white pubescence (Fig 3a). Legs reddish brown to black with white (sometimes mixed with some ochre) pubescence, especially middle and tip of hind femora, part of hind tibia, and first three tarsal segments covering with dense white pubescence.

Head slightly narrower than prothorax, closely punctured, feebly concave at vertex. Eyes deeply emarginate, inferior eye lobes slightly higher (female) or twice as high (male, Fig. 2h) as genae below it, width less than (female) or more than (male, Fig. 2h) half of frons. Antennae exceeding elytral apex in both sexes, but male is slightly longer than female; scape feebly thickened apicad, without any ridge, apex without cicatrix; ratio of the length of segments (male): 17 : 2 : 23 : 22 : 19 : 17 : 15 : 14 : 13 : 12 : 15; (female): 16 : 2 : 21 : 20 : 18 : 17 : 15 : 13 : 12 : 11 : 13.

Prothorax almost as broad as long (female) or slightly longer than broad (male), swollen laterally before middle; disc feebly convex and closely punctured.

Elytra angled at humeri, slightly narrowed apically, each with two longitudinal humeral ridges, first one beginning at humeri and not reaching to apex, second one beginning after humeri and reaching apical outer spine; apex emarginated apically, with shorter but sharp teeth at the suture, long sharp spine at the outer angle, disc with coarse and irregular punctures.

Legs stout; middle tibiae obliquely grooved ecto-apically; hind femur reaching 5th abdominal segment; 1st hind tarsal segment longer than following two segments combined in both sexes; male claws appendiculate; female claws simple.

**Figures 4–8.**
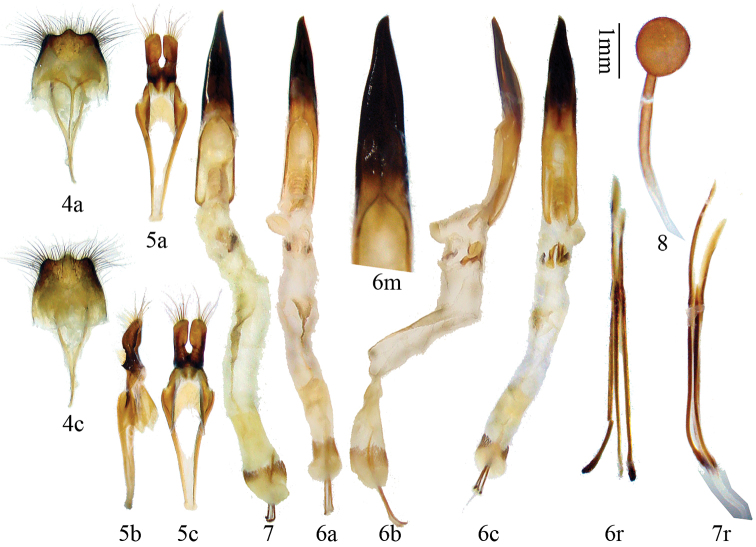
Terminalia of *Glenea jini* sp. n. **4** tergite VIII and sternite VIII & IX **5** tegmen **6** median lobe & internal sac of the male IOZ(E)1859444 **7** median lobe & internal sac of the male IOZ(E)1859443 **a** ventral view **b** lateral view **c** dorsal view **r** rods of endophallus (not to scale) **m** magnified, showing apex of ventral plate of median lobe curved to right in ventral view (not to scale) **8** female genitalia, only showing spermathecal capsule (not to scale). Scale 1 mm.

Male genitalia (Figs 4–7): Tegmen approximately 3.4 mm in length; lateral lobes can be divided into two parts (Figs 5a–5c), basal 1/3^rd^ thinner, with fine soft hairs in ventral groove (Fig. 5b), apical 2/3^rds^ expanded in three directions (dorsal, ventral and inner), with apex obliquely rounded (in both ventral and outer directions) and with fine setae which are shorter than lateral lobes; ringed part elbowed in the widest portion, converging; basal piece bifurcated distally; median lobe plus median struts slightly curved (Fig. 6b), longer than tegmen in length; the median struts less than half of the whole length of median lobe; dorsal plate shorter than ventral plate; apex of ventral plate (Figs 6a, 6m, 7) pointed, apex a little sharp and curved to right side (in ventral view); median foramen (Fig. 6m) slightly elongated; internal sac approximately three times as long as median lobe, with 3–4 pieces of basal armature, two bands of supporting armature and three unequally long rods; the longest rod approx. 1.8 mm, roughly half the length of tegmen, the middle rod shortest. Ejaculatory duct single (Fig. 7r). Tergite VIII (Fig. 4c) broader than long, apical margin tri-lobed, of which the middle one is slightly longer than lateral ones; setae around sides slightly longer than the middle ones.

Female genitalia (Fig. 8): Spermathecal capsule with a slender basal stalk and a rounded apical orb, stalk more than twice the length of apical orb.

#### Diagnosis.

Differs from other species of the genus *Glenea* by the complicated black and ochre markings and some characters of the genitalia, especially the three unequally long rods of the endophallus and the shape of the lateral lobes of the tegmen in the male. It resembles *Glenea diversimembris* Pic in the color of the pubescent markings, and the apex and humeral longitudinal ridges of the elytra, but differs in having annulate antennae; elytron with two transverse wavy vittae just behind middle; apical margin of tergite VIII (male) tri-lobed; apex of ventral plate of median lobe curved to right side (male); spermathecal capsule with a slender basal stalk and a rounded apical orb (spermathecal capsule of *Glenea diversimembris* without such rounded apical orb).

It resembles *Glenea pallidipes* Pic in the apex and longitudinal humeral ridges of the elytra, apical margin of tergite VIII (male) with a median protruding lobe, apex of ventral plate of median lobe curved to right side (male), spermathecal capsule with a slender basal stalk and a rounded apical orb; but differs in having annulate antennae; elytra with two transverse wavy vittae just behind middle; apical margin of tergite VIII (male) tri-lobed (without such lateral lobes in *Glenea pallidipes*); the slender basal stalk of spermathecal capsule not as curved as that of *Glenea pallidipes*.

From the color pattern, this species somewhat resembles *Dystomorphus* species (*notatus* Pic, *esakii* Hayashi, *piceae* Holzschuh), but differs in lacking lateral tubercles on the prothorax, the elytra bearing two longitudinal humeral ridges instead of one, and the elytral apex having a long and sharp spine at the outer angle, and so on.

#### Etymology.

The specific epithet is dedicated to Mr. Gentao Jin, a good collector, who has collected many specimens for IZAS and SHEM.

#### Distribution.

China: Tibet.

#### Type material.

Holotype: China: Tibet: male (14.0 mm long), Mêdog, Hanmi, alt. 1100–2100 m (IZAS, IOZ(E)1859443). Paratypes: China: Tibet: 1 male, Mêdog, Xirang, alt. 600–700 m, 1981.IX.25, leg. Yinheng Han (IZAS, IOZ(E)1859444); 1 male, Mêdog, Baibung, alt. 940 m, 1979.VI.4, leg. Gentao Jin & Jianyi Wu (SHEM 24207072); 1 female, Mêdog, Baibung, alt. 1000 m, 2011.VIII.10, leg. Wenxuan Bi (CCCC); 1 female, Mêdog, Dexing, alt. 980 m, 1980.V.31, leg. Gentao Jin & Jianyi Wu (SHEM 24203410); 1 female, Mêdog, Dexing, alt. 900 m, 1980.VI.2, leg. Gentao Jin & Jianyi Wu (SHEM 24204637); 1 female, Mêdog, Kabu, alt. 1030–1670 m, 1980.V.11, leg. Gentao Jin (SHEM 24204617).

## Discussion

Most of the saperdine species were historically described based only on external characters, without any genitalia characteristics being provided (Breuning 1952, 1954, 1956a, 1956b, 1958a, 1958b; Gahan 1889, 1897). An identification key for the genus *Glenea* including this species is not included in the present paper because the genus needs further taxonomical revision including genital features of the other species. However, the description of this new species provides sufficient information to allow identification and differenctiation from similar species. The morphological details of male genitalia and high quality figures presented here support the identification of the species. Preliminary studies by the first author (unpublished data) separate this species from all known saperdine species from Oriental region.

We were surprised to observe the three unequally long rods of the endophallus in this species. According to the first author’s study on saperdine beetles from Oriental regions, even when there are three rods of the endophallus, usually two equally long rods form one pair (Lin et al. 2006, 2008; Lin et al. 2009a, b, c; Lin and Yang 2011b), or three rods are subequal in length (Chou et al. 2010; Lin and Lin 2011; Lin and Yang 2011a). Additionally, in the tribe Phytoecini, when there are four rods of the endophallus, they usually form two pairs (Lin and Yang 2012). Ehara (1954) did not mention any species with three unequally long rods in his comparative anatomy of male genitalia in 101 cerambycid species from Japan.

## Supplementary Material

XML Treatment for Glenea jini
